# Impact of discussion on preferences elicited in a group setting

**DOI:** 10.1186/1477-7525-4-22

**Published:** 2006-03-29

**Authors:** Ken Stein, Julie Ratcliffe, Ali Round, Ruairidh Milne, John E Brazier

**Affiliations:** 1 Peninsula Technology Assessment Group, Peninsula Medical School, Universities of Exeter and Plymouth, Exeter, EX2 5DW, UK; 2 University of Sheffield, UK; 3 National Coordinating Centre for Health Technology Assessment, University of Southampton, UK

## Abstract

**Background:**

The completeness of preferences is assumed as one of the axioms of expected utility theory but has been subject to little empirical study.

**Methods:**

Fifteen non-health professionals was recruited and familiarised with the standard gamble technique. The group then met five times over six months and preferences were elicited independently on 41 scenarios. After individual valuation, the group discussed the scenarios, following which preferences could be changed. Changes made were described and summary measures (mean and median) before and after discussion compared using paired t test and Wilcoxon Signed Rank Test. Semi-structured telephone interviews were carried out to explore attitudes to discussing preferences. These were transcribed, read by two investigators and emergent themes described.

**Results:**

Sixteen changes (3.6%) were made to preferences by seven (47%) of the fifteen members. The difference between individual preference values before and after discussion ranged from -0.025 to 0.45. The average effect on the group mean was 0.0053. No differences before and after discussion were statistically significant. The group valued discussion highly and suggested it brought four main benefits: reassurance; improved procedural performance; increased group cohesion; satisfying curiosity.

**Conclusion:**

The hypothesis that preferences are incomplete cannot be rejected for a proportion of respondents. However, brief discussion did not result in substantial number of changes to preferences and these did not have significant impact on summary values for the group, suggesting that incompleteness, if present, may not have an important effect on cost-utility analyses.

## Background

Cost-utility analysis is regarded as an important element in the formation of policy on the use of health technologies. Guidelines suggest that a community perspective should be taken in estimating utility weights to apply to health states in decision analytic modelling [1,2]. Multi-attribute utility scales for which population weights are available provide the basis for one approach to obtaining such values [3-6]. We are investigating another: the establishment of a standing group of non-health professionals who value health states, described in short vignettes, as required by analysts. The first phase of this project was carried out with a small group of people who met in person to carry out health state preference valuation. This provided an opportunity to investigate the impact of sharing initial preference values and allowing group discussion of the scenarios. This issue is important for several reasons and has been subject to little empirical study.

Choice theory [7] is based on several axioms, amongst which is that an individual's preferences regarding any bundle of goods are complete i.e. that the process of elicitation merely reveals preferences and does not, of itself, influence the preferences [8]. Under such an assumption, variation in preferences measured at different times is only a function of measurement error. While the axiom of completeness may not be important for the validity of microeconomic theory [9], a different situation may pertain where the goods of interest are health related [10].

Evidence for lack of completeness could be counted as evidence against the theory's validity. Fischhoff has suggested that preferences are not complete but are developed and clarified by trial and error i.e. that the process of elicitation is an integral part of a process of preference development, rather than a neutral method by which an already complete preference is measured [10].

Empirical evidence for incompleteness has been reviewed by Ryan and San Miguel [11]. Preference reversal in response to minor framing effects in the presentation of preference elicitation experiments suggests that the elicitation procedure may play an important role in forming responses [12-15]. In the literature examining willingness to pay, environmental economists have noted, using a range of terms, people who do not have formed preferences [16].

The practical importance of the completeness axiom, as pointed out by Shiell et al [17], lies in its implications for the accuracy of efforts to measure strength of preference. If preferences are not complete, and develop during and beyond the process of elicitation, then the apparent variance in values from a group of individuals will underestimate the true variation in values. This has potentially important implications for the use of preference data e.g. in decision analytic models as utility weights to calculate cost per QALY. If preferences are incomplete, then use of such values without some form of correction will underestimate parameter uncertainty in the model, and may (though not invariably [18]) have important impacts on policy decisions.

To our knowledge, the impact of group discussion immediately after individual and independent elicitation of preferences has not previously been studied. We therefore tested the hypotheses that facilitated discussion in a small group would result in (a) no changes by individuals and (b) no significant changes in the summary utilities from the group.

## Methods

The group was recruited from Non-Executive Directors of local healthcare organisations (Primary Care Trusts), contact with the local voluntary sector and by advertisement in a local newspaper. Non-Executive Directors are members of the Boards of local (NHS) healthcare organisations. Part of their role in that capacity was to bring a lay perspective to the business of the organisation. The mean age of participants was 64 years (range 51 to 80 years, SD 7 years). Table 1 shows other summary characteristics of the group.

**Table 1:** Panel characteristics

**Panel Characteristics**		**N (%)**
Sex	Male	9 (60)
	Female	5 (40)
Marital status	Married	13 (87)
	Widowed	2 (13)
Occupation	Retired	8 (53)
	Working part-time	7 (47)
Level of educational attainment	Higher degree (MSc, PhD etc.)	7 (47)
	First Degree (BSc etc.)	2 (13)
	NVQ Level 4–5, HNC, HND	2 (13)
	2 or more A' Levels	2 (13)
	Other qualifications	1 (7)
	Data missing	1 (7)
Ethnicity	White	15 (100)

Participants were familiarised with the standard gamble technique in one to one training sessions, lasting around an hour, with a refresher session at the beginning of each group meeting. The group met five times over six months, with each meeting lasting about three hours. Meetings began with a reminder of the standard gamble task and presentation of the scenarios that were to be valued. It was emphasised to participants that it was not intended that the group reach a consensus and there was no obligation to take the views of others into account in considering each scenario after discussion.

41 health state scenarios were developed by one of us (KS). 35 described different severities of six conditions, which were developed from disease specific outcome measures, and six were derived from the EQ5D – a generic preference based measure of health status. Scenarios were presented to participants in "table" format [20] and were not labelled with the name of the condition being described. The conditions depicted during the study were multiple sclerosis, Gaucher's disease, osteoarthritis of the hip, Crohn's disease, eczema, and heart failure i.e. a mix of common and rare conditions. The standard gamble procedure was carried out using a top-down titration search procedure, recorded using a paper form. Individuals carried out this initial preference measurement task independently i.e. no discussion or sharing of responses was permitted. When all participants had completed the task, the preferences elicited were fed back to the group and a short period of discussion followed. In this, participants were invited to comment on the scenario and the reasons for their responses in the standard gamble task. No time limits were set on the discussion, although the facilitator (KS) intervened when it appeared the discussion had left the subject or no further comments were forthcoming. Participants were then given the opportunity to revise their responses in the standard gamble response sheet.

We noted the number of times preference values were changed, the values before and after discussion and the impact changes had on the range and summary measures (mean and median) from the group. Utility values before and after discussion were compared using paired t-test and Wilcoxon Signed Rank test. Possible associations between personal characteristics and likelihood of making changes to initial utility values were investigated using X^2 ^and t-tests as appropriate.

After the five meetings were complete, we carried out semi-structured telephone interviews with each of the participants. We asked participants "What do you think of the discussion time after each scenario?"; "Do you feel that you have sufficient discussion time?" "How helpful is the discussion and why?". Interviews were recorded and transcripts read and re-read by two of us (KS and TC). Emergent themes were identified independently and then compared and discussed, according to the principles of grounded theory, although we did not carry out concurrent analysis and data collection [21]. Nevertheless, a list of themes, constituting a preliminary analytical framework arising from the data is described.

## Results

441 responses to the 41 scenarios were collected from the group. Table 3 shows the number of changes made and differences in preferences following discussion. At least one change was made in each meeting of the panel. Sixteen changes in responses were made (3.6%) to fourteen scenarios. One individual changed five responses, out of a total of 26 responses by this participant made during the study (19%). One participant made three changes (9% of their total responses) and three made two changes (8.0%, 7% and 5% respectively of their total responses in the study). The remaining two participants made only one change (6% and 3% of total responses). The difference between individual preferences before and after discussion ranged from -0.025 to 0.45. Two thirds of the changes were positive. The average change to an individual's response was 0.042.

**Table 2:** Individual changes made to preferences after discussion

**Panel Member**	**N scenarios considered**	**N (%)changes made**	**Differences (after – before discussion)**
A	25	2 (8.0)	-0.125, 0.05
B	30	2 (6.7)	0.05, 0.025
C	41	2 (4.9)	0.45, -0.075
D	16	1 (6.3)	0.2
E	35	3 (8.6)	-0.025, 0.1, 0.05
F	34	1 (2.9)	-0.05
G	26	5 (19.2)	-0.025, 0.075, 0.025, -0.1, 0.05

**Table 3:** Summary preference values before and after discussion

		**Before discussion**		**After discussion**		**Difference**	
**Health state**	**Description**	Mean (SD)	Median (range)	Mean (SD)	Median (range)	Mean (SD)	Median
1.	Multiple sclerosis state 1	0.75 (0.21)	0.85 (0.48–1.0)	0.83 (0.16)	0.88 (0.48–1.0)	0.08 (-0.01)	0.03
2.	Multiple sclerosis state 2	0.94 (0.06)	0.98 (0.85–1.0)	0.93 (0.05)	0.93 (0.85–1.0)	-0.01 (-0.05)	-0.05
3.	Gaucher's disease	0.88 (0.14)	0.93 (0.5–1.0)	0.88 (0.14)	0.93 (0.5–1.0)	0 (0)	0
4.	EQ5D state 1	0.97 (0.03)	0.98 (0.9–1.0)	0.98 (0.02)	0.98 (0.93–1.0)	0.01 (-0.01)	0
5.	EQ5D state 2	0.3 (0.51)	0.48 (-0.88–0.88)	0.30 (0.51)	0.48 (-0.88–0.88)	0 (0)	0
6.	EQ5D state 3	0.75 (0.19)	0.83 (0.38–0.98)	0.75 (0.20)	0.83 (0.38–0.98)	0 (0.01)	0
7.	Heart failure state 1	0.84 (0.26)	0.93 (0.05–0.98)	0.84 (0.26)	0.93 (0.05–0.98)	0 (0)	0
8.	Heart failure state 2	0.51 (0.42)	0.56 (-0.68–0.93)	0.50 (0.41)	0.56 (-0.68–0.93)	-0.01 (-0.01)	0
9.	Moderate eczema	0.782 (0.22)	0.88 (0.28–1.0)	0.786 (0.22)	0.88 (0.28–1.0)	0.004 (0)	0
10.	Severe eczema	0.70 (0.23)	0.68 (0.3–1.0)	0.69 (0.21)	0.68 (0.3–0.95)	-0.01 (-0.02)	0
11.	Osteoarthritis state 1	0.96 (0.06)	1.00 (0.83–1.0)	0.96 (0.06)	0.98 (0.83–1.0)	0 (0)	-0.02
12.	Osteoarthritis state 2	0.83 (0.13)	0.83 (0.58–1.0)	0.83 (0.11)	0.83 (0.68–1.0)	0 (-0.02)	0
13.	Osteoarthritis state 3	0.96 (0.04)	0.98 (0.88–1.0)	0.97 (0.03)	0.98 (0.93–1.0)	0.01 (-0.01)	0
14.	Osteoarthritis state 4	0.98 (0.02)	0.98 (0.93–1.0)	0.98 (0.02)	0.99 (0.93–1.0)	0 (0)	0.01 (0)

Changes in utility affected the group's range of responses to a scenarios in only four instances. The impact on summary values was limited. The median for the group changed in only four cases (range of differences in medians -0.05 to 0.03). The group mean was very slightly affected in all cases (range -0.01 to 0.08) but in only one case was the difference before and after discussion greater than 0.01. The average effect of discussion on the group mean was an increase in utility of 0.0053. No differences before and after discussion were statistically significant.

Personal characteristics of participants were not associated with the likelihood of making changes to initial utility values.

The qualitative element of the study showed the group were unanimous in finding discussion helpful. Group members recognised that few changes were made in response to the discussion, but nevertheless valued this part of the process highly. We identified four themes in their responses.

### 1. Reassurance about personal preferences

The discussion period provided an opportunity to reflect on initial preferences and provided reassurance about the individual's initial response.

"But it can help you to reinforce or indeed it can clarify areas of doubt which you may have had"

*"Because depending on the view of the panel members, ... I think it reassures people, it reinforces the basic ideas*"

### 2. Procedural performance

The discussion allowed the group to reflect on the processes undertaken and to ensure that they maintained the appropriate assumptions regarding, particularly, perspective and health state duration and consistency. This was not only perceived as an opportunity for the group to «correct» aberrant responses revealed during discussion, but the knowledge that the discussion would take place appeared to concentrate the minds of respondents on the task in case they were found by the group to have carried out the procedure «wrongly».

"When you don't discuss things you could get people putting in things... because they are not having to justify or talk about it ... I think the really important thing is actually discussing it and having to justify what you've written"

"Sometimes those people who are a bit off line – like we occasionally get somebody who says, 'oh I was thinking about that from another point of view' then we all chime in and say, 'ah, but you're not supposed to do that'. And somebody on their own could easily get off key a bit without anybody realising it"

### 3. Increase group cohesion

The group reported enjoying the meetings. The discussion period after initial preference elicitation was seen as an important part of the group's interactions which, in turn, was a reason for maintaining attendance.

"It makes people relax, it's good for people to talk. It makes everybody more of a group"

"It's feeling part of a team and the feeling that you are achieving something important"

### 4. Satisfy curiosity about how others come to their decisions

The group were clearly interested in each others' perspective on the health state and valued the discussion as a means of satiating curiosity about how and why people reached their preference.

"What I enjoy is seeing where other people are coming from and noticing how their personal agenda comes into it"

"I think it's nice to hear what people, why people made their decisions"

## Discussion

We found that a brief period of discussion of the scenarios and initial preference values resulted in few changes to values, although a substantial proportion of the group (40%) made at least one change during the course of the study. Importantly, the impact of changes made at the group level, even in this small group, was negligible. Despite this, members of the group rated the discussion period as very important, for four main reasons: providing reassurance about initial preferences, checking procedural performance, increasing group cohesion and satisfying curiosity. We were interested in the impact of discussion and group cohesion. We hypothesised that, if discussion was important to people in formulating their preferences, then the physical meeting of the group may be a valuable feature. Our findings suggest that discussion may be important to maintaining interest in the group and adherence to the task.

Our study has a number of strengths and weaknesses. Utility estimation is generally undertaken as a solitary exercise, regardless of the method of data collection (face to face, telephone, postal or internet). We are not aware of any other studies which have investigated eliciting preferences individually in a group setting and explored the impact of sharing information and attitudes. Although the group was very small, 41 scenarios were valued and over 400 preference estimates were subject to potential change as a result of discussion. Nevertheless, statistical power was low in the individual comparisons and the possibility of a type II error in these analyses remains relatively high. For example, in the case of the largest difference in means before and after discussion (0.08), power was no more than 0.56. Despite this, the size of differences are such that we think it unlikely that they would have important impacts on cost-utility analyses. This hypothesis cannot be explored, however, without further research in the context of real decision problems.

A further limitation is the relative homogeneity of the participants. All were white and relatively well educated and therefore do not represent the population at large. There is therefore a case for further research into the impact of discussion, and additional information, in a more diverse population.

We are not aware of any studies which have examined the effect of discussion on preferences elicited in a group setting. Dolan *et al *studied the impact of discussion in a focus group setting [19] but this examined theoretical health care purchasing decisions and not preferences on health states. An effect of discussion was shown but the choices made by participants were subject to a wide range of considerations which do not enter into preference elicitation in the context examined here.

Evidence regarding the completeness of preferences comes from only a few studies.

Several authors have examined the reliability of preference elicitation techniques and this provides some indication of the stability of preferences over time. However, it is not possible to state whether evidence of poor reliability is a function of the nature of the measurement tool or as a result of the formation of preferences over time. Feeny *et al *described the test-retest reliability of the standard gamble in a sample of people with hip osteoarthritis who rated their own health and three marker states up to four times [22]. Test-retest reliability coefficients were moderate and ranged from 0.49 to 0.62. There was no evidence of an effect of time between ratings.

Shiell et al, in a study of repeated preference elicitation on two health states in 42 people showed that, for most of the sample, preferences were stable over repeated testing, suggesting completeness [17]. However, one-third of the group changed their responses and suggested that the interview process had prompted them to think about their values more deeply. We found a similar proportion of people changed their responses, although the frequency of changes was low. Like Shiell et al, the impact on summary measures was limited.

There are some important methodological differences between our study and this study by Shiell et al repeated elicitation after 7 weeks to reduce the impact of recall on subsequent testing. They reported that a significant number of people reported events happening in the interim which affected their values, although their results were not substantially affected by the exclusion of this group. It is not a requirement of expected utility theory that preferences are stable throughout life. We allowed reflection immediately after elicitation and therefore the impact of intervening events was removed.

Shiell et al and, in a commentary on this paper, Oliver [23] highlight the potential role of familiarity with the elicitation procedure as contributing to variability in utility values. This was unlikely to be important in our study as the group were familiar with procedures prior to collection of the data presented here, although it is possible that learning effects persisted into the study period.

The scenarios used by Shiell et al were presented in narrative form and were written in the third person. Preferences were therefore expressed in relation to the subject of the scenario and not the respondent. This introduces an additional source of variability in preferences since respondents had to consider, in particular the social consequences (handicap) of the health state for a third party. In contrast, we emphasised to participants that they should consider the possible impacts of the health states in the context of their own lives.

Shiell et al, in a further study, examined the test-retest reliability of standard gamble and time trade off techniques across three measurement occasions in a five week period in 92 people recruited from the Australian general public. At the individual level, there was some evidence that variation in responses fell after repeated administration of the task, suggesting that «participants came to recognise the value that they would ascribe to a health state as a result of participating in the study». The generalisability coefficient (corresponding to test-retest reliability) was 0.55, indicating reasonable reliability. There were, however, a small number of people whose values showed marked variation across the measurement period. Shiell et al found, as did we, that variation in individual measurement had no important effects on the group summary preference values. In this study, Shiell *et al *also tested the effect of encouraging reflection on values between measurements by allocating some participants to receive a booklet after the first interview which explained the nature and rationale behind the study and the questions asked and described what the participant's responses implied about the value of the state they had considered. Participants were asked to review their previous responses prior to subsequent interviews and consider whether the answers expressed previously still reflected their values in relation to the health state. There was no evidence for an effect from this intervention.

Ryan and San Miguel extended the work of Shiell *et al *by examining completeness in the context of a contingent valuation experiment [11]. They addressed the hypothesis that respondents are more likely to form preferences through elicitation where the goods being valued are less familiar by examining preferences for a supermarket, a dentist practice and a bowel cancer screening test. They investigated stabililty of preferences by carrying out the valuation task on three occasions, each separated by three weeks. Ryan and San Miguel found little evidence of incompleteness. Contrary to the hypothesis, preferences for the bowel cancer screening test appeared, if anything, to be more complete than those for the other bundles of goods. Furthermore, preferences appeared stable within and between interviews, suggesting that there was «very little evidence of the construction of preferences».

By using contingent valuation as the framework for their study, Ryan and San Miguel avoided some of the difficulties in carrying out preference elicitation using the standard gamble. Their results suggest that, in over two thirds of cases, there is evidence for the completeness of preferences. However, in one third, there was, at least, some uncertainty over completeness. In this respect, there is remarkable similarity between the results of the three approaches taken by Shiell et al, Ryan and San Miguel and ourselves.

There are several possible interpretations of our results. They may be taken as weak evidence supporting the completeness of preferences. We suggest the evidence is weak, because other interpretations are possible and it is not possible to reject the hypothesis that preferences are incomplete. Firstly, preferences may be incomplete but members of the group did not change their values because of social desirability bias i.e. they perceived that changing values was not valued by the group, and since the rest of the group were aware that a member changed values (though not the actual change made), this would be avoided. However, we have no evidence to support the presence of such a bias. The research team recall no discussion or behaviours which suggest that changes were considered inappropriate by the group and we took care to ensure that sufficient time was available during each session for changes to be made.

The second possible explanation of our results lies in the nature of the discussion. Although a detailed thematic analysis was not carried out, our general impression was that participants discussed their personal attitude to the scenarios and presented little new information to the rest of the group. That is, they described their attitude to different elements of the scenario e.g. pain versus mobility problems, or they described the impact that a condition would have on their ability to carry out activities of personal importance e.g. writing or skiing. Participants did not, for example, attempt to name the condition depicted in the scenarios – such labelling might have been expected to result in a change in values [24]. In other words, the limited influence on discussion may have been due to the type of information shared. The third possibility is that the amount of information was insufficient to influence values. However, we are confident that the discussion period, which in all cases was open-ended, reached saturation as we allowed it to continue until it ended or the group had left the subject of the scenario completely. Finally, the discussion took place immediately after the initial preference elicitation task. It is possible that a longer time of reflection may be required in order for a change in preferences to become apparent, although the other studies in this area suggest this is not the case for most respondents.

Although different methods have been used, there is important consistency between our study and those by Shiell et al, Ryan and San Miguel. In the majority of cases, there appears to be limited evidence for the development of preferences through the elicitation process and by reference to limited additional information or reflection. This appears to hold when using different methods of preference elicitation and in relation to a wide range of scenarios. Moreover, the impact of variation in preferences, which may be due to incompleteness, as well as measurement error, appears to be limited when considering the summary measures of utility arising from a group of individuals.

## Conclusion

Relatively little research has been carried out into the completeness of preferences regarding health states. Our study, and other important efforts in this area, suggest that, for a minority of people, preferences may develop during elicitation. It is not surprising that there should be some fundamental variation in this respect, given the apparent differences between people in such psychological traits as extroversion and introversion. Further work is required to explore what determines the stability or otherwise in preferences and it will be challenging to understand the influences which may arise from characteristics of study participants, the scenarios being considered and elicitation processes. Nevertheless, our study found little evidence that preferences are altered by reflection and discussion immediately after elicitation in the majority of people and, more importantly, the assumption of completeness is not unsafe from the perspective of analysts using the summary values obtained from a group.

## Competing interests

The author(s) are the project team for the NHS Value of Health Panel Project

## Authors' contributions

KS conceived the study, developed the protocol, carried out analyses and drafted the paper. JB assisted in design of the study and commented on the draft paper. JR assisted in design of the study and commented on the draft paper. AR assisted in design of the study and commented on the draft paper. RM assisted in design of the study and commented on the draft paper.
